# How sensorimotor interaction shapes and supports young children’s gestural communication around science

**DOI:** 10.1080/09500693.2021.1909771

**Published:** 2021-04-23

**Authors:** Rhiannon L. Thomas Jha, Sara Price, Minna O. Nygren, Esme Glauert

**Affiliations:** Institute of Education, University College London, London, UK

**Keywords:** Children, gestures, communication, thinking, science

## Abstract

Research has demonstrated that gesture produced during conversation can provide insights into scientific thinking and can aid scientific communication in adults and school-aged children. However, to date, there has been a limited exploration into the role of gesture in supporting young children’s science communication, and how this is underpinned and shaped by their sensorimotor experiences. This study examines, identifies and conceptualises ways in which children spontaneously used gesture during their interaction-orientated discourse and how this mapped to their action experiences at a water table. Findings show how gestural communication in children under 5 years of age can convey different levels of complexity related to science thinking.

## Introduction

Science for children in the early years may be best conceptualised as a process, where children learn through *doing* and *discourse* centred around interaction (e.g. Siry et al., 2012). As such, science provision for the early years tends to focus on providing hands-on interactive activities that utilise visual prompts, sensory objects or manipulative apparatus to demonstrate and provide the sensorimotor experiences of the principles at hand. In order to provide children with effective interactive activities these need to be designed to give children opportunities for ‘doing’ science, where the ‘doing’ is meaningfully linked to the underlying science principles, and facilitators/educators need to be open to the multiple modes with which children will engage, both during their interactions and afterwards. Children in this age group may be particularly likely to draw on multiple modes during interaction and discourse, both because their science process tends to be situated predominantly in action rather than in speech, and because their domain-specific language skills may be limited relative to their abilities to engage with science processes through interaction. In this manuscript, we focus on children’s use of gesture, as gestures are likely to draw on the particular interactive experiences that children have, and can provide insights into the role of these experiences in shaping children’s science thinking and communication (e.g. Roth & Lawless). Gesture has also been suggested to provide evidence of the embodiment of action experiences (e.g. Hostetter & Alibali, 2018), yet little research has examined pre-school children’s use of gesture linked to action experiences in their science communication.

Research in embodied cognition emphasises the importance of *meaningful* ‘bodily’ experience in learning and development. Specifically, this points to the need for sensorimotor interactions that *meaningfully* exploit action-based experiences that are instrumental in shaping the way we conceptualise the world around us. While the importance of action experiences in children’s developing cognition has been emphasised for decades (Piaget, 1970), theories of embodied cognition challenge this transitional view suggesting that action experiences are fundamental forms of knowing (Glenberg, 2010). An embodied perspective on learning suggests that action/gestural resources should not only be part of learning experiences but may also be essential for providing sensorimotoric representations that children later use to express and elaborate their ideas about these experiences (e.g. Alibali & Nathan, 2012).

A substantial body of work in embodied cognition highlights the importance of gesture as evidence of the role of bodily experience in supporting children’s thinking and communication (e.g. Austin & Sweller, 2017; Roth & Lawless, 2002), illustrating children’s readiness to learn (Goldin-Meadow, 2011), providing insight into children’s thinking (Pine et al., 2007) and aiding communication (Clark & Lindsey, 2015). This work suggests the importance of particular underlying action experiences in giving rise to the specific gestures used to explain, express or externalise ideas. ‘Specific information content in student gestures and directed actions supports meaningful insights – gestures are more than hand-waving – and must connect underlying concepts, pedagogical language, and student understanding’ (Weisberg & Newcombe, 2017, p. 2).

While this work is primarily situated in mathematical learning contexts (e.g. Goldin-Meadow et al., 2001), a small body of research suggests that gesture is a valuable tool for (older) school-aged children in their scientific conversations. Since gesture has the potential to provide insight into the ways children conceptualise scientific ideas, and in turn inform the design of science activities that are grounded in *meaningful* action, this research asks: in what ways do sensorimotor experiences underpin gesture? What role does gesture play in pre-school children’s communication and meaning making? What insights can this provide into their thinking? In so doing, we aim to illustrate the link between action, cognition and discourse, highlighting the critical role embodiment has on learning, and the importance of specific design of hands-on science learning activities paired with interaction focused discourse in the early years.

## Background

### Embodied cognition

Theories of embodied cognition emphasise the important role of the sensory body, experience, emotion and social interaction for learning and development (e.g. Alibali & Nathan, 2012; Barsalou, 2008; Varela et al., 1991). In the context of the work reported here, we draw on three key ideas. Firstly, the developmental progression of our understanding of the world is grounded in our bodily experience (physically, socially and culturally): ‘cognition depends on the kinds of experiences that come from having a body with particular perceptual and motor capacities that are inseparably linked’ (Thelen et al., 2001, p. 1). For young children’s science learning, this has implications for the design of the kinds of sensorimotor experiences that are developmentally appropriate.

Secondly, the situated nature of interaction is central to meaning making, where understanding is based and dependent on previous experience and socially/culturally mediated meanings, as well as influenced by the current context of interaction. This points to the importance of how the designed (science) experience – the concrete world – helps children to make meaningful connections both across and within the sensorimotor activity.

Thirdly, our sensory systems are inextricably intertwined with cognitive representations. In other words, in situ sensory experiences provide sensorimotoric representations – or ‘embodied tools’ – that are then used in later reasoning, and support the linking from action to abstraction (Weisberg & Newcombe, 2017). By analysing embodied representations, through actions or gestures we aim to gain insight into young children’s gestural tendencies, their meaningful communication of science ideas, their understanding of underlying science ideas and the interaction between these.

### Gesture research

Throughout this paper, we focus on spontaneous, representational gestures (Hostetter et al., 2007; Thelen et al., 2001): non-verbal behaviours (primarily produced by the hands) which tend to accompany speech, and are produced by an individual from their own impulse (without being explicitly requested or prompted by the researcher). Representational gestures are used to represent attributes, actions, and relationships of various entities, such as objects and people. These could be either iconic gestures, which refer to a concrete referent, or metaphoric gestures which refer to an abstract referent. We particularly focus on representational gestures produced by the hands (rather than deictic or beat gestures), as these are most likely to convey information about the science topic being discussed.

Children (and adults) use multiple modes of communication simultaneously to express meaning. Different modes are more or less effective at conveying certain aspects/features of phenomena (Danielsson, 2016). For example, gesture is an effective way of communicating dynamic visuo-spatial information, such as direction, shape or speed. Hostetter and Alibali (2018) propose that gesture is a form of simulated action, i.e. gestures arise from embodied simulations of motor and perceptual states. Speakers gesture because ‘they simulate actions and perceptual states as they think, and these simulations involve motor plans that are the building blocks of gestures.’ Sometimes speakers activate imagistic representations or grounded modal representations which resemble the experiences they represent. In these instances, the same neural areas involved in the action or experience are activated and under these circumstances, gestures are more likely to occur. If this is the case then we might expect gestures to closely resemble the actions and experiences in which they are grounded. Hostetter and Alibali (2018) argue that individuals can schematise particular elements of visual, spatial or motoric information within these simulations and adapt their gestures to the particular context. This suggests that actions and experiences provide the foundations for gestures as these will be drawn upon when a speaker simulates their properties.

Science/scientific thinking from pre-school onwards is centred around hands-on interactive activities that utilise visual prompts, objects or apparatus to demonstrate and provide sensorimotor experience of the principles at hand. Much of this involves observation and noticing visuo-spatial features related to action experience, and as such gesture could prove to be particularly useful at supporting science discourse when props are no longer present. However, it is also important to consider how specific science activities might foster later gesture in the pre-school age range and how these gestures might support ongoing thinking.

A few studies have explored school-aged children’s gesturing around science topics: cog motion (Boncoddo et al. 2010), balance (Pine et al. 2004), and floating and sinking (Callinan 2014). These studies identified gestures which were relatively consistent across children and could be used as evidence of understanding, for example, reference to weight, distance and the mid-point in either speech or gesture suggested children understood a balance beam task (Pine et al., 2004). Thus, when children are given a particular science activity to engage with, they might embody this experience in a relatively consistent manner and later draw on this experience to shape and aid their gestural communication.

Based on their study of gesturing by 10-year-olds talking about the seasons, Crowder and Newman (1993) suggested that gestures may play an important role in the construction of scientific insights, as some children used an interplay of gestures to help establish relationships between parts of a model, and then translated their gesture into the verbal language of the classroom. Findings suggest this interplay between speech and gesture may be at the heart of online scientific explanations as it indicates that the child is working to build, run and revise a model, rather than just recalling a memorised model. This highlights the importance of valuing discourse, supported by gesture as an ongoing scientific ‘process’ through which children can continue to make meaning.

**Figure 1.**
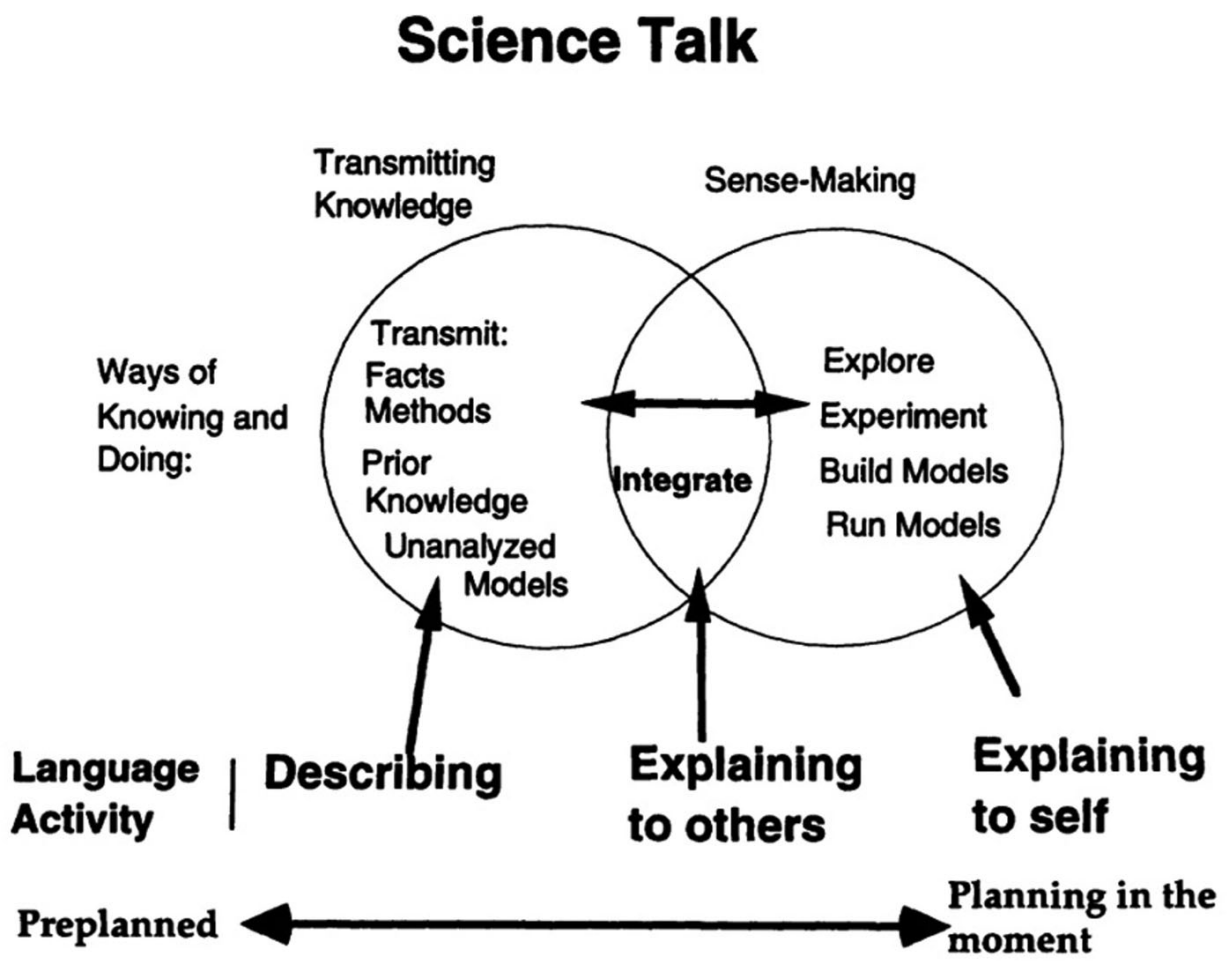
Diagram of Science talk reproduced from Crowder (1996).

In a follow-up study, Crowder (1996) suggested that science talk could be broken down into two (sometimes overlapping) types; transmitting knowledge (a passive process) and sense-making (an active process of coordinating theory and evidence and developing models). Based on video data from school children (aged 10 years) explaining what makes the seasons change, Crowder and colleagues developed a science talk schema (Figure 1), which attempted to distinguish these two types of science talk. This research suggests that gesture is both a medium, which children can use to help them engage with science ideas, and which observers use to help understand the ‘science talk’ that a child expresses.

Other work shows the benefits of multimodal discourse – that includes bodily knowledge such as gestures – into the interaction between pre-schoolers and teachers in scaffolding their science understanding (e.g. Kallery, 2011; Samuelsson, 2019). Samuelsson’s work illustrates the role of ‘doing, languaging, and the use of external artefacts and tools’ (p. 267) in the scaffolding process. Drawing on notions of embodied cognition and the role of gesture in communication, this exploratory study aimed to better understand the ways in which particular sensorimotor experiences underpin gesture, the role that gesture plays for pre-school children’s communication and meaning making, and the insights it may provide into their thinking, including the degree to which their gestures move beyond exact replicas of their action experiences and are drawn upon to highlight certain features, patterns or relationships. In so doing, we aim to extend our understanding of the relationship between action and gesture to pre-school-aged children, and the role that gesture plays in their science communication and thinking.

## Methodology

Given the limited research understanding of the role of gesture in pre-school children’s science communication, an exploratory research study design was employed to gain an in-depth understanding of how gestures give insight into how children think about and communicate their sensorimotor experiences and relevant science ideas, in particular, how they observed, described and potentially explained their object interactions with a water table.

### Participants

Twenty children aged 3–5 years (Mean = 4.6 years, 12 female) were recruited from a London nursery school. Parents gave signed informed consent for their child/children to participate. Children were invited to participate and informed that their participation was optional and that they could choose to leave the interaction at any point without consequences.

### Procedure

The study took place in a classroom attached to the nursery school, where children interacted in pairs with a water table. As the water table is a familiar activity for young children, we encouraged interaction with minimal adult intervention. Before interaction children took part in a warm-up conversation to become familiar with the researchers and the research context. After interaction children took part in a semi-structured interview conducted in one corner of the room, with children sitting on child-sized chairs facing the researcher/interviewer, but oriented away from the apparatus. A second researcher ensured that two video cameras captured the content of the interviews and interaction effectively: a primary camera captured the majority of the scene, and a secondary camera provided an alternative point-of-view where necessary.

#### Warm-up conversation

Children were asked about everyday experiences which related to water. Example questions included:

- What happens if you keep filling a cup, up and up and up?
- What happens when you jump in a swimming pool?
- Why do some children have to wear armbands?

#### Water table interaction activity

**Figure 2.**
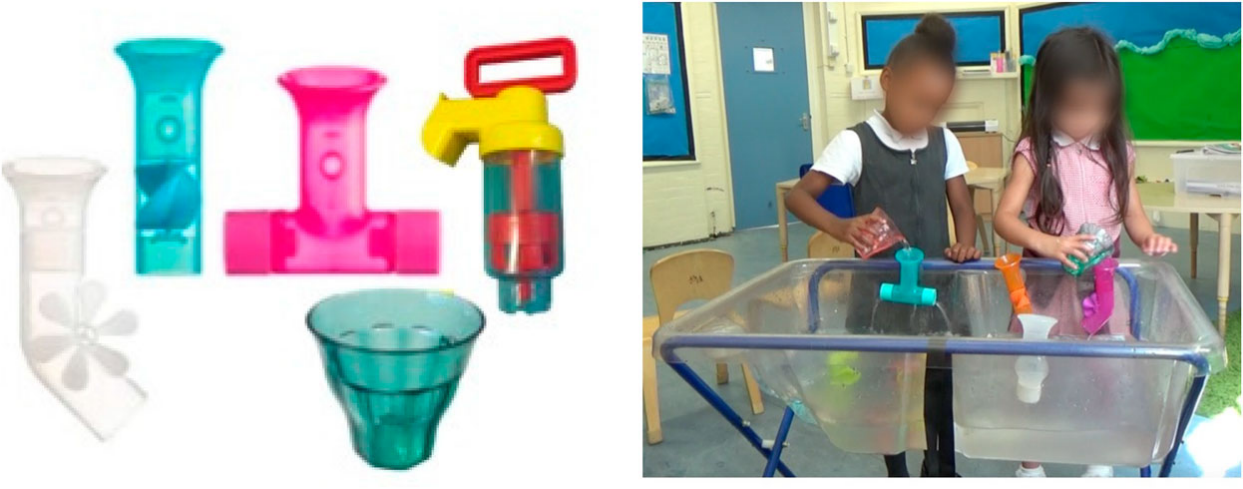
(a) Example objects used at the water table; (b) Two children interacting at the water table.

Children freely interacted with the water table and various water play objects (Figure 2(a)), which provided opportunities to experience science ideas around spatial/temporal relationships, and cause and effect, as well as science ideas linked to the particular objects, such as water flow, containment, and rotational motion (Figure 2(b)).

*Portable pump:* The pump had a transparent cylinder with a brightly coloured handle and spout. As children raised and lowered the pump handle, water was drawn through the perforated base into the cylinder and out of the spout. As the cylinder was transparent children could observe the effect of moving the handle and thus raising/lowering the attached disc. This object was designed to be used with the base submerged in water, when it was raised and rotated, water could leak out through the spout and handle.

*Tubes with waterwheels:* Two tubes with water wheels could be handheld or affixed to a surface using a sucker, children could use a cup or other object to pour water into the tubes. Affixing the tubes to the side of the water table allowed children to interact with them using both hands, but changed the way they could view the tubes. The water wheel elements protruded partially out of the tubes so that they could be spun from the outside manually or with water, as well as by pouring water into the tube.

*T-shaped tube:* The T-shaped tube had one outlet with three small holes and another with a single larger hole. This tube could be handheld or affixed to the water table, but when attached it was difficult to observe the water flowing out, due to its height relative to the child and the water level. When children held up the tube its features were more evident, as was the water flow from the tube.

*Cups:* Four semi-transparent cups in various colours, but identical in size and shape, could be used to collect water and pour it either directly into the water table or into another toy. As the cups were semi-transparent, children could see the level of water inside and could observe the water moving from the cup.

#### Post-interaction interview

After interaction children were interviewed in pairs to engage them in discourse centred around their interactive experiences. This allowed us to explore how children drew on their interactive experiences to support discourse when the physical resources were no longer available. They were asked to talk about their interaction: ‘can you tell me a bit about how you played with the water table?’, followed by questions probing descriptions they had given, or the apparatus they interacted with, for example:

- Did you play with the pump? What did you do with it? Then what happened? What made that happen?
- Did you use the different tubes? How were they different? What happened when you put water into the blue one? What made that happen?
- Did you try the pink tube? What did that one do? How did you make it spin? What made it spin?

Children were given an opportunity to ask any questions before being escorted back to their classroom.

### Data analysis

Data analysis drew on multimodality and cognitive event analysis. Cognitive Event analysis takes cognitive events/moments as the starting point for analysis and works backwards to explore preceding interactions between an actor and their environment which might have led to this event, placing focus on the ‘distributed cognitive system’, to include others, the environment, technologies and more (Steffensen et al., 2015). The ‘event’ here was a child’s representational gesture, as evidence of the way in which they had embodied their interaction experience, and drew upon this for thinking and communication. We sought to explore the children’s interaction experiences which allowed them to establish a particular embodied representation and to later use this to support their thinking and communication. Since Cognitive Event analysis focuses on Cognition, in particular problem solving, this method cannot be wholly applied to our data as children were not given a specific task or problem. Therefore, we also took a multimodal analytical approach, which emphasises situated action, focusing on recognising, analysing and theorising the different ways in which people make meaning. It examines the semiotic resources available within a specific context and people’s situated use of resources for making meaning (Jewitt, 2013), including body posture and movement, facial expressions, gestures, and speech. In so doing, it attends to embodied engagement of interaction, and embodied communication of ideas. Meaning is considered to emerge through the iterative connection between the meaning potentials of the objects, actions and outcomes available within the context of the children’s activity, and their subsequent communication around these objects and ideas. The analysis, thus, focused on how children interacted with the different resources –objects and one another – in the sensorimotor experiences, with specific attention to action, gaze, gesture and speech in relation to their activity; and how they used their body for communication, to examine the communicative resources of gesture and bodily movement related to their sensorimotor experience.

The analysis involved three key stages. Firstly, we produced multimodal transcripts for each post-interaction interview, describing the gestures, actions (rare), speech and gaze/body position observed throughout the interview, accompanied by screen-shots from videos to illustrate the gestures, actions and gaze/body positions. We then identified instances where children used their hands and/or wider parts of the body to express experiences/ideas. Sixteen of the 20 children produced spontaneous representational gestures during the post-interaction interview: a total of 208 spontaneous representational gestures with a mean of 10.4 (SD = 10.08) gestures per child. From this corpus we identified gestures which referred to the objects included in the interaction activities (cups, T-shaped tube, water wheel tubes, and pump) by considering the question which preceded the gesture, the speech which accompanied the gesture as well as the gesture itself. We take an object-focused approach rather than a science concept-focused approach, as the interactions revolved around the various objects, which can be used to explore different science-related foci.

Stage two used these post-interaction gestures to select specific object interactions for analytic focus, for each child/pair of children. For example, if a child produced a representational gesture when communicating about the pump we selected all instances where that child, or their peer, interacted with the pump for further analysis. A multimodal transcript was produced for each clip.

Stage three involved mapping between the post-interaction gestures which children produced and their interactions with the relevant objects. We examined the sensorimotor action experiences children had with particular objects, and how they seemed to draw upon these experiences during their later communication. At this stage, we collectively identified emergent themes and common observations across the corpus. These themes were iterated and refined over a series of discussions which focused on: the repertoire of gestures produced by children discussing similar science ideas; the function these gestures might have in terms of science communication and thinking; the insights these gestures might give into children’s conceptualisations; and the link between these gestures and the action experiences observed.

## Results and discussion

The results are presented in relation to gestures and action linked to the water play objects, first identifying how they are used for communication, then how they are used to support thinking/reasoning.

### Gestures for communication

#### Gestures and their underlying action experiences around the pump

Children’s gestures drew on their activity with the pump, with ten children *re-enacting* the way they handled and acted on the toy, occasionally using this re-enaction as a referent. For example, children produced a verbal label like ‘thingy’ while re-enacting using the pump to clarify which ‘thingy’ they meant, or children did not explicitly label the pump yet the referent object was evident through their gestures.

**Figure 3.**
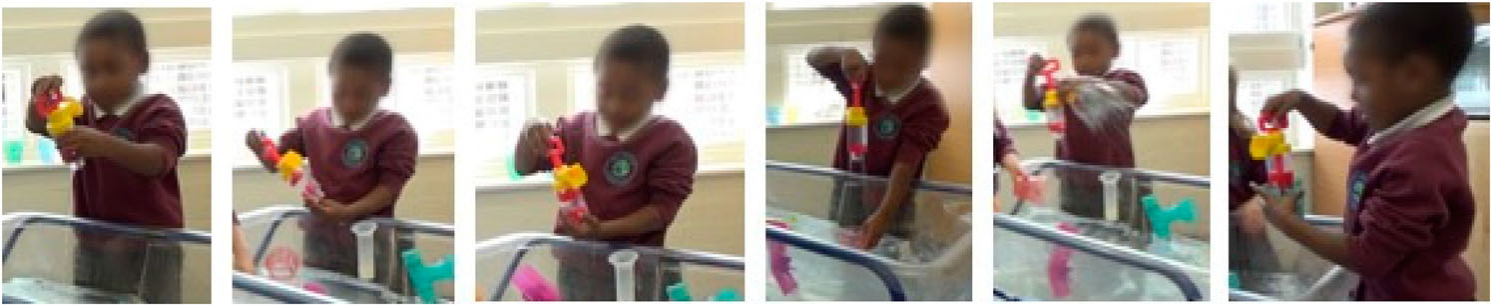
(a) P32 explores the pump by (L-R): rotating it; manipulating its features manually; enclosing the base with the palm of his hand; raising it and allowing the water to drain; generating a dramatic splash and placing the base inside a cup of water. (b) Multimodal transcript example from P32’s post-interaction interview. He draws attention to the central cylinder of the pump and how its volume changes, then adjusts his posture and gaze while he considers when the pump creates a large splash, once he has prepared an answer he turns back to the researcher and correctly clarifies that the pump functions when the handle is drawn up rather than pressed down, saying ‘when you pull it up’.

Six children produced gestures around the pump which went beyond their own action experience and captured their observations. These gestures typically *described* the water flowing/splashing out of the pump. Different children represented different aspects of this water flow in their gesture. For example, some focused on emphasising the scale of the ‘splash’ whilst others focused on the shape of the water trajectory. These gestures tended to capture features which were richly spatial and that children had visually observed. For example:

*Vignette:* P31 and P32 interacted with the pump during the same interaction session. In the subsequent interview, P31 produced a re-enactment of the pump action followed by a precise depiction (*observational description*) of the trajectory water formed as it left the pump. In comparison, P32 represented multiple different features of the water flow through the pump. He depicted the scale of the splash generated by the pump using a whole-body gesture, thus giving this feature particular emphasis. This links to his own heightened experience of the pump splashing (Figure 3(a)), where the pump produced a ‘splash’ so large that it landed outside of the water table and onto the floor. Both of these speak to concepts of water flow, illustrating how the children are making relational links between their pumping action (force) and the water trajectory. P32 also re-enacted more specific details of the actions he produced to generate this effect. Whilst most children produced relatively stereotyped ‘pumping’ enactions, P32 demonstrated through a combination of speech and gesture that the water splashes when the pump handle is brought up, rather than pushed down (Figure 3(b)). P32 also began to produce a *causal description* of the pump mechanism using gesture to depict that the size of the chamber changes as the handle is drawn up and down. P32’s interactions with the pump were extensive and his discoveries appeared to occur through accidental moments like the splash, as well as through meticulous investigation (Figure 3(a)).

Some children interacted with the pump in the way it was designed to be used, with the handle upright and the open base placed in or above water. Other children interacted with it in alternative orientations, the most frequent of which aligned with prior experience of water pistols, with the handle towards their body, the spout facing downwards and the open base pointing away. However, when children interacted with it as a water pistol, water leaked out and it was ineffective both as a pump and a water pistol. These children had distinctly different experiences of water flow related to pump interaction compared with those who played with it in an upright position. This demonstrates that even a simple manipulation has the potential to affect the sensorimotor experiences encountered through an object, and the specific foundations for future thinking and communication. Those children who used the pump as a water pistol had limited experience of the particular mechanisms of the pump, yet gained other experiences around containment, gravity and water flow. Specifically, that the pump chamber initially held the water, which gradually trickled out into the water table below. Since water pistols are themselves forms of pumps, through discourse or interaction children could be further encouraged to consider the ways in which this object did and did not function as a water pistol. More broadly, sensitivity to the meaning that children make through their interactions allows us to further support their scientific thinking, in particular through discourse grounded in their interactions. Furthermore, this highlights the importance of considering which sensorimotor experiences are foregrounded within an activity, and whether these align with the targeted science ideas – a simple manipulation like fixed or free moving pumps will significantly shape children’s opportunities for meaning making.

#### Gestures and their underlying action experiences around tubes with water wheels

Children drew on multiple modes of communication when they ‘talked’ about the water wheel tubes. Five children *re-enacted* their own actions around these, for example holding the tube while pouring water from a cup or flicking the wheel. However, nine produced gestures which went beyond this direct experience to *describe their observations* of the way water moved through the tubes and the way the wheel span as a result, suggesting their understanding of the relationship between water flow and the wheel spinning. When depicting the water wheels, children used a variety of representational gestures. Some represented the motion of the wheel using various ‘spinning’ gestures, which captured the general circular motion of the wheel, these varied in orientation (on a horizontal or vertical plane) and focused on the circular aspect of the motion.

Two children produced gestures during their discourse of the water wheels, which captured something about their notions of how and why the wheel span, suggesting an understanding of causality; P32 and P26.

**Figure 4.**
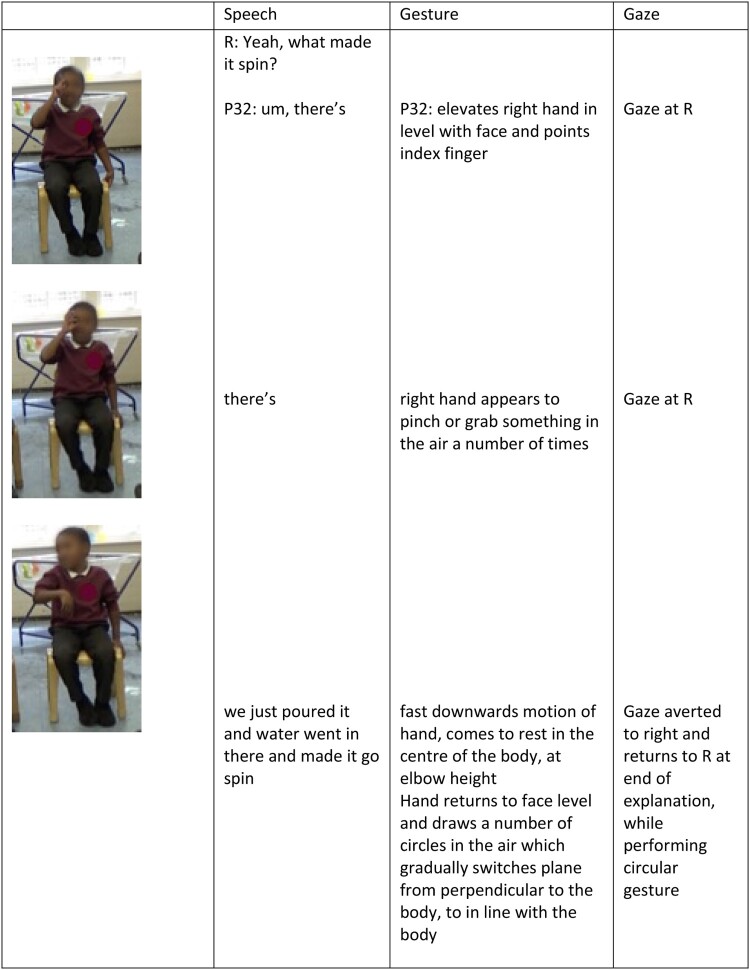
Multimodal post-interaction interview transcript, engaging with the question of what made the wheel spin.

*Vignette:* P32’s gestures centred around the central pin of the wheel, whilst he sought a *causal description* of what it was about the wheel that made it spin. Through re-enaction and in-depth description, P32 seems to be in the process of building a model of how the system works, breaking down the system and focusing on particular elements and their role in the system as a whole. In this instance, he focuses on his actions and their outcome (in terms of the way the wheel span), presented across speech and gesture. However, through his language, but in particular his gestures (Figure 4), it was clear that he was in the process of generating an explanation of this phenomena and was fixated on one of the salient features of the mechanism – the fact that the wheel was centrally fixed and therefore had to spin around this point.

**Figure 5.**
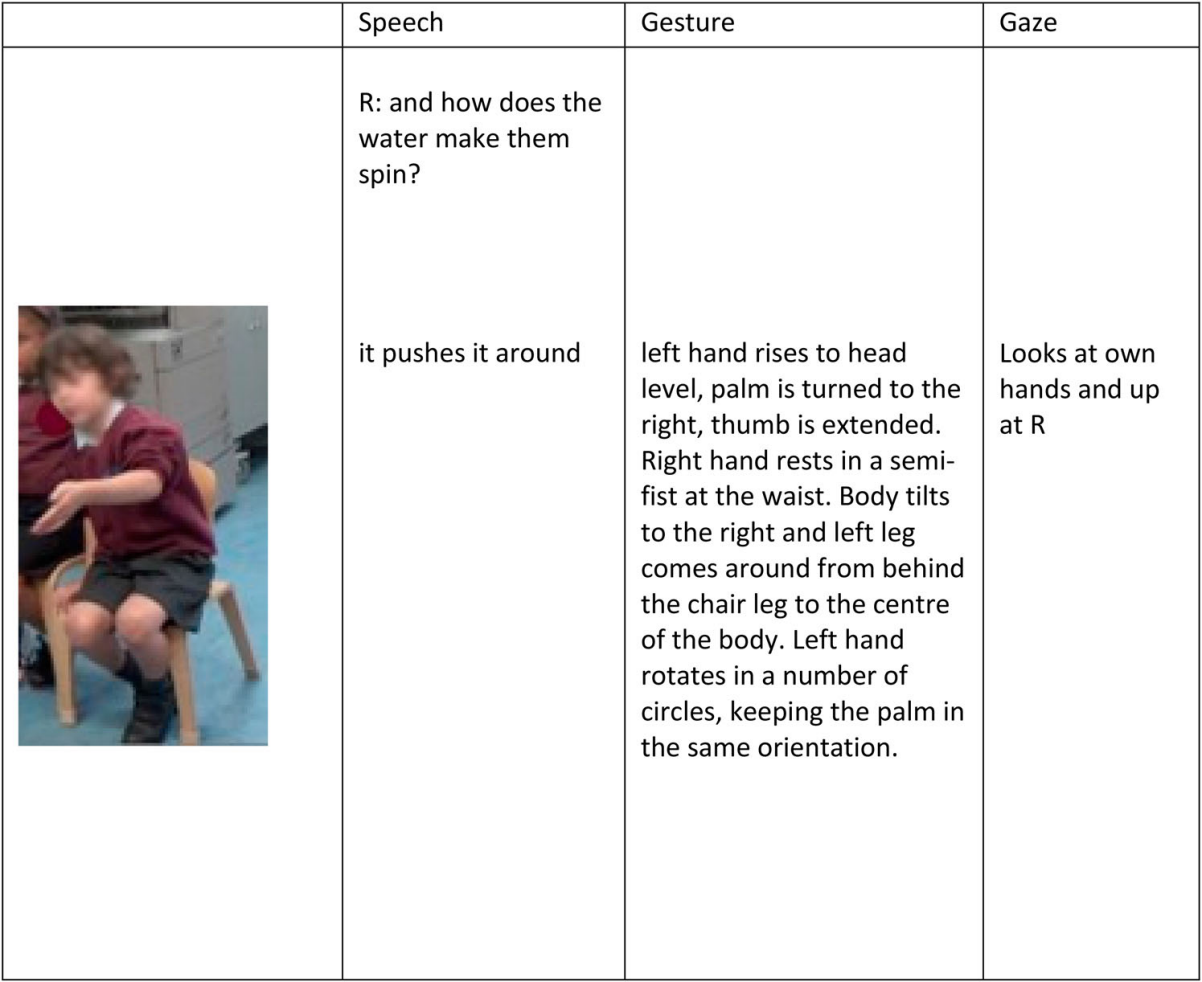
Multimodal transcript extract from P26’s post-interaction interview.

P26 focused on the blades of the wheel and produced a *causal description* of how the wheel span, stating that the water ‘pushes’ the wheel around whilst demonstrating through gesture that this ‘push’ was focused on the angled blades of the wheel and this led to the circular motion of these blades (Figure 5). This gesture separated the blades of the wheel, from the ‘whole’ of the system and drew particular attention to essential features of this component, which cause the wheel to turn (surface and angle).

Through language and in particular gesture, it is evident that this child identified one of the relevant features which underlie the phenomena of spinning – the fact that there are blades which have a particular angle and that water hits the surface of these blades and produces a push force. Here he is engaging with the relationship between water flow and rotational motion of the blades, and the underlying causal mechanisms of the blade positioning.

#### Gestures and their underlying action experiences around the T-shaped tube

The T-shaped tube drew children’s attention to the particular way that water flowed through and out of the tube. In their discourse, four children produced re-enactions around this object, for example showing how they submerged the tube to scoop up water. Eight children used gesture to support their *observations* of how water flowed through the tube. One child used gesture to support a *cause and effect explanation* of why water flowed through this tube in a particular way, linking this to its shape. The gestures which children produced linked back to the particular interactions they had, for example:

*Vignette*: P18 and P19 both poured water through the T-tube while they held it above the level of the water. P19 initiated interactions with this tube and repeatedly submerged it and lifted it to allow water to flow through, whilst imploring P18 to ‘look, look!’. After a number of repetitions P18 reached out and touched the water flowing from the tube. Later, P18 explored the tube by holding it whilst water drained out, and slowly rotated it on a horizontal plane, while she touched the water with her other hand. She also submerged this tube together with another straight tube with small holes in its base, filling them both with water, and then holding them aloft, observing the water flow from each tube.

**Figure 6.**
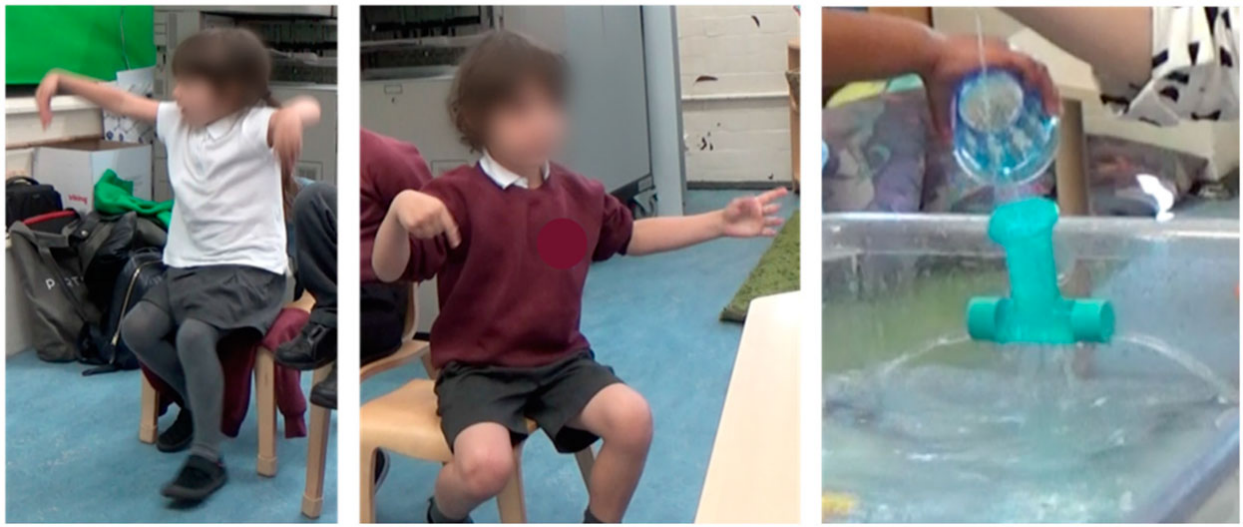
P18 (left) and P26 (centre) using gesture to describe how the water flowed through the T-shaped tube (right).

During these interactions, both children expressed interest and excitement around this particular tube. In their later conversation, P18 drew on this experience and gave a description of the water flow through this tube, using gesture, speech and bodily movement. She used her body to represent the tube, her arms showing how the water flowed out horizontally in two directions. She also used her body as an analogy, stating that the tube had shoulders, supporting this with a gesture which originated at her shoulders, and then depicted the water flowing out and down (Figure 6). Here her gestures drew particular attention to the flow of water she had observed as well as the shape of the tube, linking these ideas together In so doing she illustrates a causal link between the specific pattern of water flow and the shape of the tube and drew links between this structure and the structure of her own body.

P26 also repeatedly submerged and lifted the T-shaped tube carefully observing each end and commenting that there was ‘more than one stream of water’ during his interaction. Similarly, to P18 he represented the two ‘arms’ of the tube using his arms, but in contrast, he used gesture to draw particular attention to one ‘arm’ producing a single stream, whilst the other ‘arm’ produced multiple streams (Figure 6). P26 produced an *observational description*, through his gesture, of how the water flowed from the tube, suggested an understanding of the relationship between water flow and tube construction.

P26 and P18 had similar experiences with the T-shaped tube, leading them to produce superficially similar gestures. However, these children differed in their particular noticings, one which focused on the ‘bigger picture’ of how the water flowed versus the details of the flow.

#### Gestures and their underlying action experiences around the cups

Nine children re-enacted their actions with the cups using gestures that were highly consistent: holding their hand in an open c-shape with thumb upwards, as if holding the cup in their hand. They would then move their arm, whilst maintaining this hand posture to demonstrate how they collected water in the cup, transported it and poured it.

**Figure 7.**
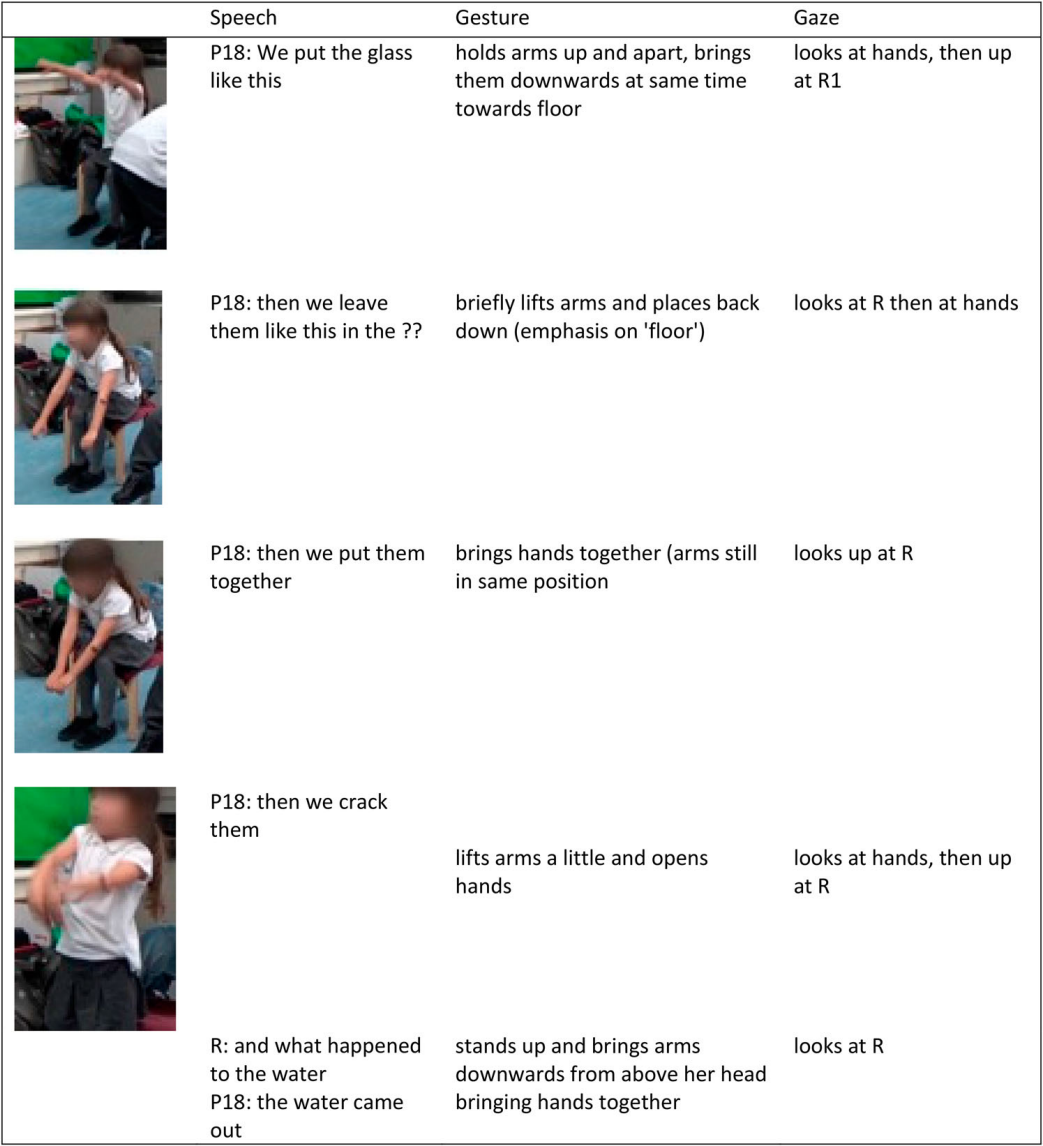
Multimodal transcript extract demonstrating P18 using a combination of speech and gesture to re-enact actions involved in her ‘game’.

Some children developed imaginative uses of the cups and these diverse experiences were later drawn on during conversations. For example, P18 and P19 devised a game whereby each child held two cups, submerged them under water sideways and then attempted to lift them while maintaining a seal between the cups. Once the cups were raised above the water the children would break this seal, releasing the water trapped inside. Later P18 re-enacted this experience in a step-by-step manner, as if giving instructions on how to play the game (Figure 7). She drew on an ‘egg’ analogy, stating ‘then we crack them’, concluding this re-enaction by describing her observation of the flow of water out of the cups using her whole body.

Other children used the cups in combination with the pump, placing the base of the pump into a cup of water, highlighting the fact that water was being taken up from the cup into the pump base, as well as drawing attention to the volume of water being moved. Whether the volume of water is limited and whether the system is open or closed in an activity can help draw attention to water flow and the activity of the pump.

This further demonstrates how the activity children engage with shapes the science ideas which may be foregrounded through their actions and, in turn, this will shape their meaning making and embodied discourse.

### Gestures for thinking and reasoning

We have illustrated some ways in which gesture is used in young children’s communication, which in itself can support their thinking and reasoning. Some examples of gesture for thinking and reasoning may be more explicit than others. For example, (Kim et al., 2011, p. 208) state ‘It seems they *[children]* simply gesture by and for themselves … the students’ bodies, through gesturing, enact and bring forth emergent forms of knowing.’ Within our corpus, we identified moments of gesture which were in line with this interpretation. Similarly to Kim et al. (2011), in these moments, children oriented their upper body away from their communicative partner, averted their gaze and their speech became fragmented. Using an example reported above, we draw particular attention to the way that the child seems to be using gesture to support his own thinking. Here both gesture and speech become fragmented as ideas were initiated and then seemingly abandoned before being fully articulated in either mode. While these gestures are still visible and may play an important role in communication, the speaker provides a number of cues to suggest this is not their intent.

*Vignette:* Through gesture, P32 appears to be holding and manipulating the spinning wheel as he considers what made it spin. He begins and aborts speech in conjunction with these gestures (e.g. ‘there’s … there’s … ’) supporting the notion that he is thinking-through this mechanism. However, this thinking-through appears to primarily happen through his hands as his speech does not capture his thinking until later in the vignette. At first, he extends his index finger, potentially in relation to the fixed centre of the wheel. He then produces and appears to abort two gestures which ‘grasp’ at the wheel in some way. Finally, he produces a fluid explanation in speech, which is directed at the researcher and seems to resort back to a more superficial schematic description (observational), accompanied by relevant gesture (Figure 4). This process of thinking-through gesture appears to suggest that P32 is trying to build a model of the system he is describing, in which he is linking his own actions and the subsequent observations he made, whilst trying to propose a hypothesis of what particular feature if the system leads to a particular outcome (spinning).

Figure 3(b) also shows how P32 uses adjustments of his posture and gaze to indicate that he is continuing to think about the problem at hand. This is a powerful conversational cue, which allows a child to moderate the speed of conversation and give themselves space and time to think, which may be particularly valuable in an educational setting – especially if the topic being discussed is complex and adults may be quick to conclude that a child is struggling and move on. Furthermore, his use of his body may be fundamental to the way that he is able to think through this idea, as he uses his hands to manipulate the problem and to build a model of the system he previously interacted with.

## General discussion

Three key contributions emerge. Firstly, this extends previous work on gesture for communication in older children and adults to show its value for younger pre-school children’s science communication. Secondly, it provides further evidence that thinking and communication are grounded in bodily experiences. Thirdly, it identifies different gestural functions in relation to science ideas for children under 5 years of age.

### Gesture for science communication in pre-school children

Similar to previous research (e.g. Boncoddo et al., 2010; Callinan, 2014; Pine et al., 2004), we found some consistency in the gestures children used when they were discussing the particular aspects of activity, specifically to re-represent the objects they had interacted with (re-enaction). Children’s gestures to evoke the pump tended to be bimanual, at approximately waist height and involved re-enacting the motion they used to make the pump work. Similarly, children used handling/modelling gestures to evoke various objects they had interacted with and these tended to be closely linked to the initial experience and, as such, relatively consistent across children. This builds on previous work by extending these findings to younger children and demonstrating that children may produce quite consistent gestures when their sensorimotor experience was equivalent.

However, when it came to conveying science ideas, which went beyond the action experiences themselves, these gestures began to diverge, and appeared to show distinctions in not only how different children conceptualised these science ideas, but also how they differently embodied these ideas. For example, some children demonstrated water flow with precise finger movements, whilst others used their whole bodies to capture similar motion. This may be partly because their exact experiences were influenced by small differences in their own actions in relation to the objects. For example, whilst multiple children used the basic function of the pump (raising and lowering the handle to move water), some children placed the base of the pump in the water table, some held it aloft, some rotated as they pumped, some placed the base in a cup of water, some did a mixture of these activities. Typically, when children came to describe the pump system they re-enacted the action of raising and lowering the pump handle; however, from this point, their observational and theoretical descriptions varied depending on their particular interaction and, within that interaction, which features they identified as salient. This poses an interesting dilemma when considering the design of science activities, as on the one hand this variability could be controlled by fixing the location of the pump giving children a more uniform experience (likely producing less variability in their gestures), however on the other hand this variability gives children the potential to have multiple experiences from a single object which overtime is likely to help them produce a more complex and complete model of the system and how it works. This extends previous work by foregrounding the role that sensorimotor experiences have in shaping young children’s subsequent gesture. In turn, this highlights the importance of considering the activities children engage with, and how these translate science into action. Changes to the activity such as whether parts are loose or fixed, whether they are part of an open or closed system, how they are positioned relative to a child, the materials they are made with (e.g. transparent vs. opaque, light vs. heavy) will shape the sensorimotor activities children engage with, which in turn will shape meaning making both in the moment and in later multimodal discourse.

The repertoires of gesture elicited extends previous findings, which typically focused on small subsets of gestures aligned with a narrow science activity, for example, cog-tracing in the context of cog motion (Boncoddo et al., 2010). This is not surprising since these activities were restricted in terms of the nature of the action experiences that children could have, and the science ideas which were evoked. In contrast, children in our study were free to interact with the objects as they wished with no particular task, eliciting a broader range of experiences. Even when children had superficially similar experiences their attention was differentially drawn to particular features.

A novel finding was the variation in gesture use across children, specifically when they were discussing complex science ideas, which spoke to the underlying mechanisms and relationships within a system. These included demonstrating the spatial relationships between events, for example showing how their actions with the pump led to water moving in a particular way, and showing the mechanistic elements of an object: or that the angled blades of the water wheel were pushed by the water. This finding is both valuable and challenging for early years science settings. It is challenging as this variability may make it more difficult for educators to readily identify and understand the content of children’s science gestures. However, it is useful as these gestures give a true insight into the underlying models of relationships and mechanisms that children are constructing and considering in relation to these complex ideas. This creates an exciting opportunity to use gestures as evidence for very young children’s complex scientific thinking. Gesture provides children with a spatial and dynamic resource, which they can use to help reason about complex systems in the absence of other resources, and which provides some insight into the way they think.

### Gesture as re-enaction, observational description & causal reasoning

Of the sixteen children who produced spontaneous representational gestures, thirteen produced at least one gesture which *re-enacted* their activity. Most occurred during multimodal science discourse, however occasionally they used as referents in the absence of a verbal label for an object/action. These gestures re-represented children’s action with a high degree of accuracy, to the extent that the re-enacted instances could easily be identified from their gestures alone. This links to the claim that the developmental progression of our understanding of the world is grounded in our bodily experience (Thelen et al., 2001). In line with Roth and Lawless’s study (2002) of high school children developing scientific understanding and Crowder’s theory of model building through gesture (1996), we propose that these re-enactions provide evidence that pre-school children create embodied models of physical phenomena they have experienced, and these models can be evidenced in their gestures.

Thirteen children also produced at least one gesture which went beyond ‘activity’ to *describe their observations* and the result of their actions within the broader context. These *observational descriptions*, captured the links between children’s own actions and the outcomes of these actions, for example ‘I poured the water and then the wheel span’. This process of engaging in science discourse embedded in action allows children to break down the system into parts and to focus on particular features which they feel are salient. This combined process of re-enacting/re-engaging with their own actions and observations may help emphasise temporal and spatial features of the phenomena and make causal relationships between events more apparent. The fact that this process can be evidenced in children’s gestures suggests that the children’s body-based experiences will shape their continuing exploration of these ideas, beyond the limits of the interactive activities themselves.

Two children produced at least one gesture which supported their *descriptions* of how their actions, the objects and the outcomes were linked and why particular actions might have led to particular outcomes. Although this is a small proportion of the total number of participants, it is an important preliminary finding, as it demonstrates the potential for complex gestures around science in this age range. Observing and predicting are fundamental aspects of the scientific process, here we show that children can communicate their observations and focus on particular variables of importance in their multimodal discourse, giving them the potential to further engage in the scientific process, with support. Furthermore, it provides evidence of an internal process of embodiment which may be occurring for a broader sample of the children, but may not be evident in their gesture. Identifying the gestures which children use to support their discourse of cause and effect relationships could help to further develop and support interactive science activities for this age range.

This account extends work with much older children to pre-school children, progressing from being grounded in simulations supported by actual objects and moving towards abstractions (Roth & Lawless, 2002). Interestingly, in Roth and Lawless’s study students were supported by the teaching resources throughout their learning, that were seen as a key part of their development from sensorimotor iconic gestures to symbolic iconic gestures, to verbal utterances. In contrast, children in our study were not supported by any of the resources underpinning these ideas during their ‘science talk’, yet despite this, they were able to produce descriptions which went beyond their own actions and drew out complex relationships and salient features underlying these relationships.

As such, it is particularly important that we consider the design of children’s sensory motor experiences, as these will be precisely drawn upon when children re-enact and describe their experiences. Some models of adult gesture (e.g. Hostetter & Alibali, 2018) suggest that even if children do not produce gestures which demonstrate the embodiment of these sensory motor experiences, they may still be re-engaging with these experiences internally. If, as we suggest, these re-enactions/re-engagements allow children to begin to make and express relationships between phenomena in a scenario, then it is essential that these relationships are apparent in the original sensorimotor interactions, and the actions required are meaningfully aligned with the underlying concepts. For example, if children operated a pump by pressing a button (e.g. in digital interactive spaces) then they would not experience the relationship between their own particular pump actions and the impact this had on the flow of water and the moving elements of the pump. Furthermore, if children draw upon this button pressing action in later science talk then the effectiveness of this gesture as a referent is limited since the action and the dynamics of the system are not explicitly mapped to one another, and as this action is common across various contexts, is likely to carry less meaning.

From our analysis, three levels of complexity of expression (description) emerged across the gestures children produced related to their prior interaction experiences. These gesture categories move from a focus on one’s own actions, to being able to describe additional observations about objects and their features and finally to being able to link one’s own actions, relevant contextual features and outcomes. We suggest that the process of producing these kinds of gestures (or potentially engaging in discourse designed to promote these gestures) might not only capture something about the understanding a child has, but might also allow them to build on this understanding through use of gesture as an ‘embodied tool’ (Weisberg & Newcombe, 2017). We see this as part of the process of ‘doing’ science, whereby engaging in discourse around these activities and supporting this discourse through gesture is in and of itself part of the process of doing science and further shapes children’s thinking. Thus, when considering the design of science activities for young children emphasis should be placed on the sensorimotor experiences offered by the activities, how these actions convey particular science ideas, and how best to engage children in discourse which values gesture and helps them to build on their action experiences through embodied reasoning.

## Conclusion

Three key contributions emerge from this work.

Firstly, findings extend previous work on gesture for communication in older children (e.g. Roth and Lawless, 2002) to show that pre-school children also effectively utilise gesture as part of their science communication, and in ways which go beyond simply reproducing their own action experiences. It seems that gesture is a valuable tool for young children to draw upon for science communication as it allows them to: (i) substitute verbally unknown referents or descriptors with gesture, (ii) convey rich visuo-spatial information in a compelling and succinct manner, (iii) re-experience and newly explore their science experiences.

Secondly, we provide further evidence for the embodied cognition paradigm that thinking and communication are grounded in bodily experiences, evidenced by the particular action experiences children encountered being directly drawn upon in their subsequent science communication.

Finally, we provide evidence that pre-school children’s gestural communication around science can take on different levels of description. Through engaging in discourse around their science activities children can re-explore these interactive experiences within the context of this discourse. Re-enactions of their own actions provide evidence that when engaged in science discourse children can draw on and re-engage with their physical experiences in the absence of the physical objects. This is evidence that interactive activities can provide children with an ‘embodied toolkit’ for thinking and communication. Furthermore, gestures which went beyond their own actions to capture their observations, suggests that interactive activities can support children’s ongoing science processes by providing them with embodied resources, which enable them to begin to construct models of the systems at hand, drawing particular attention to spatial and temporal relationships between actions, features and outcomes. If the actions that children have engaged with are meaningfully linked to the underlying science ideas then they can make the causal relationships in a system transparent allowing children to engage with these relationships and later reproduce them in speech and gesture.

By exploring instances where children demonstrate evidence of their ideas around cause and effect in their gestures, we have the potential to inform the design of interactive activities that support pre-school children’s multimodal discourse and communication – beyond speech – which might foster children’s development of these embodied models. By supporting children to engage in discourse focused on ‘doing’ we might support them to continue to develop their science processes, whilst also giving them the opportunity to express current thinking. Aligning with Samuelsson (2019) this highlights the importance of the educator both in the initial design of activities which provide children with meaningful ways to ‘do’ science and in providing a discourse context which can support children’s continuing processes of meaning making.
